# The Impact of Persisting Hyperactivity on Social Relationships: A Community-Based, Controlled 20-Year Follow-Up Study

**DOI:** 10.1177/1087054712436876

**Published:** 2014-01

**Authors:** Jaime Moyá, Argyris K. Stringaris, Philip Asherson, Seija Sandberg, Eric Taylor

**Affiliations:** 1 King’s College London, UK; 2 Royal Free and University College Medical School, University College London, UK

**Keywords:** ADHD, adult ADHD, cohort study, epidemiology, relationship quality

## Abstract

**Objective:** The purpose of this study was to examine whether persisting hyperactivity into adulthood was associated with impaired family, friendship, and partner relationships or poor coping skills in everyday life. **Method:** A 20-year community-based follow-up of 6- to 7-year-old boys showing pervasive hyperactivity (*n* = 40) and unaffected controls (*n* = 25) was conducted. At age 27 years, participants were assessed with detailed interview techniques as well as self-report ratings. **Results:** ADHD in adulthood was associated with problems in intimate relationships and negotiation skills. Antisocial behavior did not influence the association, but remitting childhood hyperactivity was not associated with social relationship difficulties in adulthood. **Conclusion:** In an untreated, community-based sample of hyperactive children, the risk for unsatisfactory social relationships is largely confined to those patients who still show ADHD in adulthood. The majority of patients who experience childhood hyperactivity have positive social relationships in adulthood.

The persistence of ADHD into adulthood has become the focus of widespread research attention, and ADHD is currently considered a life span condition (Faraone et al., 2000; Wilens & Dodson, 2004). It has been suggested that as many as 60% of childhood cases may continue with significant ADHD symptoms as adults (Biederman, Mick & Faraone., 2000; Rasmussen & Gillberg, 2000; Weiss, Hechtman, Milroy & Perlman, 1985). However, estimates of the proportion of children with ADHD who will have persisting symptoms vary considerably as a function of reporting source, attrition rate, and the criteria used to define the disorder in adulthood (Mannuzza, Klein, & Moulton, 2003).

There are several long-term prospective longitudinal studies on the persistence of ADHD into adulthood in the literature. Table 1 summarizes these studies. Databases and published articles were searched for longitudinal studies on ADHD, hyperactivity, and attention deficit. To be included, studies needed to be based on at least 30 participants; use control groups; select participants using standardized criteria, generally *DSM* criteria; retain at least 50% or more of their original samples into adulthood (above age 18 years); give details on participants lost to follow–up; and have a low attrition rate.

**Table 1.** Prospective Studies of ADHD Into Adulthood.

		Sample size	Age at baseline	Diagnostic criteria		Age at follow-up	Years of follow-up	ADHD persistence		
Investigators name and year of publication	Location of study	*n*	*M*	Baseline	Follow-up	*M*	*M*	*n*	%	Control group
Weiss, Hechtman, Milroy, and Perlman (1985)	Montreal (Canada)	61 children (59% PR) 90% boys	6-12 years	Not stated	*DSM-III*	25.1 years	15	Symptoms^a^ 42 66%		41 children aged 25.2
Mannuzza Klein, Bessler, Malloy, and Lapadula (1993)	New York (USA)	91 White boys (88% PR)	9.3 ± 1.4 year	*DSM-II*	*DSM-III DSM-III-R*	25.1 ± 1.3 years	16.1	Full ADHD 7 8% Impairment^b^ 10 11%		95 boys aged 25.6 ± 1.6
Mannuzza, Klein, Bessler, Malloy, and Lapadula (1998)	New York (USA)	85 White boys (82% PR)	7.3 years	*DSM-II*	*DSM-III DSM-III-R*	24.1 years	17.0 year	Full ADHD 3 4% Impairment^b^ 0 0%		73 boys 24.1 years (94% PR)
Rasmussen & Gillberg (2000)	Göteborg (Sweden)	55 children (42% boys)	7 years	*DSM-III*	*DSM-IV*	22 years	15 years	28	58%	46 children (43% boys)
Barkley, Fischer, Smallish, and Fletcher (2002)	Wisconsin (USA)	158 hyperactive children with 87% males (93% PR)	4-12 years	*DSM-III-R*	*DSM-III-R*	20.8 years	13.8 years	differences depending on reporting source and definition of disorder C		81 control (92% males) 90% PR
Biederman et al. (2006)	Boston (USA)	140 children (80% PR)	6-18 years	*DSM-III-R*	*DSM-IV*	21.6 years	10 years	78	70%	120 (88% PR)

Note: PR = participation rate at follow-up; *DSM-III* = *Diagnostic and Statistical Manual of Mental Disorders* (3rd ed.); *DSM-II* = *Diagnostic and Statistical Manual of Mental Disorders* (2nd ed.); *DSM-III-R* = *Diagnostic and Statistical Manual of Mental Disorders* (3rd ed., rev.); *DSM-IV* =*Diagnostic and Statistical Manual of Mental Disorders* (4th ed.)

a Weiss et al. (1985) reported persisting core symptoms in adults (restlessness, poor concentration, impulsivity), not persisting full diagnosis.

b Mannuzza et al. (1993) used the concept of “probable diagnosis” to define those cases in which not all criteria were met but functional impairment was present.

c Barkley et al. (2002) found that parents reported much higher rates of persistence than patients (66% vs. 12%) and demonstrated that the definition of disorder (developmentally appropriate criteria vs. *DSM* criteria) influenced persistence rates as well.

As apparent from Table 1, the risks associated with ADHD appear clear. However, most of the existing follow-up studies (except the Swedish one) are based on patients referred to clinics and treated, so a poor outcome could be associated with factors leading to referral, such as parents’ ability to cope with child behavior, severity of disorder, coexistent problems, school relationship problems, as well as family and social background (Sayal, 2006; Wolf & Wasserstein, 2001)—rather than the presence of hyperactivity per se. To fully understand the natural history, there is a need for more prospective, epidemiologically representative studies of untreated hyperactive children.

One purpose of epidemiology is the completion of the clinical picture. Hyperactive behavior is more common in childhood than the diagnosis of ADHD (Taylor, Sandberg, Thorley, & Giles, 1991). To develop public health strategies, it will be desirable to know whether those with hyperactive behavior are at risk for later mental health problems even if they do not meet all the diagnostic criteria for ADHD.

The nature of any deficits in adult life also needs fuller understanding. Distinguished research on the adaptive functioning of adults with ADHD has stressed that their problems extend to poor academic grades, low engagement with further education, involvement with the juvenile justice system, and risky behavior, especially in driving (Barkley, Fischer, Smallish, & Fletcher, 2006). Some degree of uncertainty remains about the full long-term impact of hyperactivity (National Institute for Health and Clinical Excellence [NICE], 2006). Clinical decisions could be better founded if there was more knowledge of social functioning of affected adults, and particularly of their ability to relate to significant others.

Social relationships are an important determinant of the quality of life, and need to be understood as part of the natural history of disorder. It has been shown that clinic-referred adults with ADHD have poorer marital adjustment and family functioning (Eakin et al., 2004), as well as a higher incidence of separation and divorce, than normal controls (Biederman et al., 2006). Furthermore, it has been demonstrated that participants who have been hyperactive in childhood have fewer close friends and report more problems with keeping friends compared with controls (Barkley et al., 2006).

In a previous article from this prospective epidemiological study (Taylor, Chadwick, Heptinstall, & Danckaerts, 1996), it was suggested that childhood hyperactivity was a risk factor for development over the period from 7 to 17 years, even after allowing for the coexistence for conduct problems. This suggested a developmental pathway through which hyperactivity raised the likelihood of impaired social adjustment. Another study based on the current data set indicated that childhood hyperactivity predicts poorer mental health in adulthood (Stringaris et al., 2011).

Taking the previously mentioned findings into account, we decided to examine social relationships in a never-medicated sample of adults, 20 years after they were ascertained as showing high levels of hyperactive behavior, and hypothesized that childhood hyperactivity would lead to disturbed social relationships in adulthood.

## Objectives

The following were the specific aims of the study:

1. To document the extent to which adults who had been hyperactive in middle childhood had significantly different levels of satisfaction in family, friendship, and intimate relationships, as well as impaired ability to negotiate, by comparison with those not hyperactive in their childhood
2. To examine whether continuing presence of hyperactivity in adulthood influences the level of satisfaction in family, friendship, and partner relationships, and the ability to negotiate
3. To examine whether antisocial behavior influences the possible effect of hyperactivity on relationships in adulthood

## Method

### Participants (Childhood Study)

The original survey, from which all the participants were taken, has been previously described (Taylor et al., 1991). As a brief summary, the participants included all 6- and 7-year-old boys (3,215 boys), on the registers of mainstream schools in the London Borough of Newham, with the schools for severely learning disabled excluded.

The Rutter B(2) questionnaire was completed for 99% of the children by their class teachers, and the A(2) questionnaire for 80% by the parents. In total, 2,462 had both screening questionnaires completed. Hyperactivity was defined as present if the teacher and parent questionnaire gave a score of 3 or more on the Hyperactivity subscale. Conduct problems were defined as present if the score on the teacher scale was 9 or greater, or that on the parent scale was 13 or greater, and the score on the Conduct Disorder subscale was greater than that for “emotional disorder.” These cutoffs had been validated in previous surveys and on the Isle of Wight studies (see Rutter, Tizard, Yule, Graham, & Whitmore, 1976).

On the basis of the screening questionnaire ratings, three groups were selected: those with scores above cutoff for conduct disorder who also met criteria for pervasive hyperactivity (*mixed*; constituting 5.3% of the study population), those who met criteria for pervasive hyperactivity but not for conduct problems (*hyperactive*; amounting to 3.7%), and those not meeting criteria for either condition (*control*). The cases were stratified by behavioral group and then randomly sampled from the resulting groups, to give approximately equal numbers in each group for detailed study. Participants were excluded if they had scores of 5 or greater on the Emotional Disorder subscale of either the parent or the teacher scale. With respect to their hyperactive symptoms, the children with mixed hyperactivity and emotional symptoms were similar to those with hyperactivity only (Taylor et al., 1991). The children with mixed hyperactivity and emotional symptoms (15% of children with hyperactivity and 12% of controls had a score of 5 or greater on the Emotional Disorder subscale) were excluded because they were considered to form an etiologically distinct group, which could have confounded the comparisons. For this reason, the results of the present study should not be generalized to hyperactive children with comorbid anxiety or depression.

## The Follow-Up Study

### Participants (Adulthood Study)

For the present study, 121 participants (79 hyperactive, 42 control) were initially selected because on the basis of their childhood behavior, they fell into either the hyperactive or control groups, and had been included in the detailed second wave of study. Of these, the follow-up of the children of first-generation immigrant families (16 hyperactive, 9 controls) will be reported separately. The marked differences in the way they were identified by parents and teachers by comparison with children of native British families, made it inapproprite to use the same criteria of identification (Sonuga-Barke, Minocha, Taylor, & Sandberg, 1993). The sample for follow-up, therefore, consisted of 63 children with hyperactive behavior (*mixed* and *pure hyperactive* combined), and 33 control children with no detected behavioral problems. Those who only met criteria for conduct problems were not included in the present analyses.

Of the 96 adults, 10 could not be traced, and 12 had refused permission for future contact when approached in a previous 10-year follow-up (Taylor et al., 1996). Of the remaining 74, 1 had died (of meningitis) and 8 declined to be interviewed. The participants reported here, therefore, included 40 hyperactive cases (63% of the original target group) and 25 controls (76% of the original controls).

### Procedure

A first follow-up was carried out 9 years after the second stage of the original survey, when the participants were aged 16 to 18 years (Taylor et al., 1996). The second follow-up reported here was carried out when the participants were 25 to 30 years old. The follow-up was based on the groups who had received detailed study.

Tracing the participants was undertaken by a variety of means, including previously recorded addresses, electoral records, and personal contacts. The reliance on multiple methods was intended to reduce the bias that might be introduced by those who fail to be contacted by any one technique. When the young men and their families were contacted, permission was sought for interviews and a test session. A small sum of money was paid to them in recognition of the expenses involved. Informed consent was obtained before interviewing the participants.

### Outcome Measures

1. Adult Functioning Interview is an investigator-based standardized interview schedule, administered to the participant by a trained interviewer. It is a modification of the Adult Personality Functioning Assessment (APFA; Hill, Harrington, Fudge, Rutter, & Pickles, 1989). The APFA interview itself includes investigator-based structured enquiry on aspects of psychosocial adjustment, including relationships with friends, partners, and family members, and ability to negotiate in social situations. These relationship measures are the subject of the present report. The interview also includes information about occupational and psychiatric outcomes: These will be the subjects of a separate report (Stringaris et al., 2011). It inquires about functioning over a period of 5 or 10 years, depending on a particular interpersonal domain. Pervasive dysfunction according to the APFA has been shown to be associated with the diagnosis of personality disorder (Hill, Fudge, Harrington, Pickles, & Rutter, 2000). In the present study, three additional behavioral scales were included: those of (a) hyperactive (inattentive/restless) behavior, (b) defiant/antisocial behavior, and (c) emotional disorder symptomatology, especially anxiety and depression. These three scales are in the format of the Parental Account of Children’s Symptoms (PACS) interview (Chen & Taylor, 2006), which involves enquiring about behavior in specific situations by trained interviewers (in the present study psychology or social science graduates). Audiotaped interviews were used to make detailed behavioral ratings on the basis of the participant’s recollections of recent behaviors in particular situations.

Reliability checks were carried out throughout the investigation by another researcher listening to and rating randomly selected tapes. The interrater agreements in terms of kappas were .74, .81, .79, and .67 for overall satisfaction with friendships, negotiation skills, partner relationships, and family relationships, respectively.

2. Psychiatric outcome was measured by the Schedule for Affective Disorders and Schizophrenia (SADS) interview, and the cognitive function assessed by Cambridge Neuropsychological Test Automated Battery (CANTAB; these are subjects of separate reports). Algorithmic diagnoses were generated from the SADS. Telephone or postal enquiry was made where possible from the men’s parents.
3. A diagnostic conference was held about each study participant by the research team (chaired by an experienced psychiatrist, E. T.), which was blind to the person’s childhood hyperactivity levels. All information available from the participants and informants concerning their interviewer and self-rated adjustment over the year prior to the assessment was systematically presented for *Diagnostic and Statistical Manual of Mental Disorders* (4th ed.;*DSM-IV*; American Psychiatric Association, 1994) diagnostic judgments. All members of the research team remained blind to the findings of previous assessments until the diagnosis had been recorded.

### Analysis

Mean differences between the hyperactive and nonhyperactive groups as defined in childhood—and, within the previously hyperactive, between those who shared ADHD at outcome and those who did not—were compared by analysis of variance and by analysis of covariance with antisocial behavior as a covariate. Where a scale was dichotomized at the level of those showing “good” function, chi-square comparisons were made.

## Results

### Sample Characteristics

On average, the participants were 27.6 years old (*SD* = 1.2) and had 0.5 children (*SD* = 0.8); 83.4% were in paid employment, whereas 13.3% were either unable to work, in full-time education/training, or caring for others (3.3% missing data). The average age of leaving school was 16 years (*SD* = 1.1), leaving home 20.5 years (*SD* = 3.6), and starting employment 16.9 years (*SD* = 2.4).

The level of attrition described earlier under “Participants” section led us to consider whether the 65 children followed up in adulthood were representative of the original 96 in terms of their characteristics at the outset of the study. Table 2 describes IQ, socioeconomic status, and level of behavioral problems for the 65 children who were followed up, and compares them with the 31 children lost to attrition. No systematic differences were found.

**Table 2.** Comparison of Those Followed Up and Those Not Contacted on Baseline Measures.

	Hyperactive			Control		
	Followed up *M* (*SD*)	Not interviewed *M* (*SD*)	*p*	Followed up *M* (*SD*)	Not interviewed *M* (*SD*)	*p*
*n*	40	23		25	8	
IQ	100 (14.8)	97 (17.1)	.39	103 (14.2)	102 (8.9)	.90
CRS	12 (4.7)	13 (4.8)	.11	2.3 (2.8)	4.1 (2.2)	.10
B2 Total	14 (6.9)	12 (6.4)	.39	4.2 (3.3)	6.0 (3.7)	.19
A2 Total	15 (5.9)	14 (7.5)	.47	9.5 (5.2)	8.8 (4.8)	.73
SES	4.1 (1.0)	4.5 (0.9)	.08	3.8 (1.1)	4.6 (0.8)	.09

Note: IQ: score on four subtests of Wechsler Intelligence Scale for Children-Revised (WISC-R); CRS = score on hyperactivity items of Conners’ Classroom Rating Scale; B2 Total = sum of behavioral problems from Rutter B(2) teacher rating scale; A2 Total = behavioral problems from Rutter A(2) parent rating scale; SES = socioeconomic status from Registrar-Generals classification of occupations (range = 1-6, 1 denoting highest occupational level). The table shows the mean scores and standard deviations on variables measured at the first contact with the project, at age approximately 7 years; according to whether contact was achieved at follow-up.

### Comparison Between Adults Identified as Hyperactive in Childhood and Their Controls

When the participants identified as hyperactive in childhood and the controls were compared for age, employment status, number of children, age leaving school, and starting employment, no statistically significant differences in any of these aspects were found. By age, those designated hyperactive (*M* = 27.7, *SD* = 1.1) were only slightly older than the controls, *M* = 27.4, *SD* = 1.5; *t*(63) = 3.76, *p* > .5, and had left home at a younger age (*M* = 20, *SD* = 3.7) than did the controls, *M* = 21.3, *SD* = 3.6; *t*(45) = −1.21, *p* > .5. There was no significant difference regarding the employment status, χ^2^(3) = 1.5, *p* > .5. The key measure for social relationships is the overall level of satisfaction with friendships. To arrive at this rating, several specific aspects were inquired about: Several aspects of social relationships were measured, such as joint activities and levels of discord with friends. In the sample as a whole, 55% reported having four or more friends with whom they engaged in joint activities and 52% had two or three friends they could confide in. The overall perceived level of satisfaction with friendships was significantly correlated with the number of friends with whom the participant engaged in joint activities (*r* = .45, *p* < .001), had a confiding relationship (*r* = .57, *p* < .001), and had received practical help from (*r* = .54, *p* < .001), as well as with the participant’s own level of sociability (*r* = .40, *p* < .001). For each friendship, the interviewer inquires further about the quality of relation to arrive at the judgment of overall satisfaction. There was no significant effect of persisting hyperactivity with regard to the overall level of satisfaction with friendships (*F* = 2.22; *df* = 1, 63; *p* = .14; Table 3) and those with a “good” level of satisfaction were as common in the previously hyperactive as in the controls, χ^2^(1) = 1.7, *p* = .19.

**Table 3.** Comparison of Relationship Satisfaction Levels Between Participants Who Had Been Hyperactive at Age 7 and Those Who Had Not (Regardless of Their Levels of Hyperactivity at Age 27).

	Childhood hyperactivity	Controls	*p*
Satisfaction with friends	2.67 [0.500]	2.87 [1.088]	.59
Family support	2.38 [0.744]	1.77 [0.898]	.87
Ability to negotiate	3.67 [1.000]	2.97 [0.983]	.69
Satisfaction in partner and intimate relationships	4.00 [0.707]	3.13 [1.332]	.70

When the degree of support the participants received from and gave to other members of their family was examined, it was found that a majority (81.2%) reported their families giving them practical and emotional support. A third stated that they were supported by both of their parents, as well as siblings; 28.1% had received support only from their mothers and siblings, whereas the rest either received support only from by their mothers or did not receive support from anyone in the family. As shown in Table 3, there was no statistically significant difference between previously hyperactive and controls on the overall level of support from the family (*F* = 0.62; *df* = 1, 58; *p* = .80) and those with a “good” level of support were as common in the previously hyperactive as in the controls, χ^2^(1) = 5.2, *p* = .27.

Another aspect examined was the participants’ ability to handle negotiations, defined as the ability to secure rights and obtain their goals using discussion, assertiveness, or even humor, instead of aggression and discord. Again, those identified as hyperactive in childhood did not differ significantly from controls in their level of ability to negotiate (*F* = 2.44; *df* = 1, 63; *p* = .12; Table 3) and were no less likely to be “good” negotiators, χ^2^(1) = 0.25, *p* = .62.

Regarding partner and other intimate relationships in the sample as a whole, the average age the participants began dating was 15.2 years (*SD* = 2.4), and the first steady relationship was at 17.8 years (*SD* = 2.8), which on average lasted for 27.8 months (*SD* = 31.2). At the time of the assessment, 41% had no current relationship, 21.7% were in a steady relationship, 35% had been in a cohabiting relationship/marriage for more than 6 months and the rest were in shorter lasting cohabitations. Among those 56.7% with cohabitations lasting more than 6 months, 20% had cohabited with more than one partner, 37% with one partner only, and 10% had never had a steady relationship. Concerning the level of satisfaction in partner and intimate relationships, as shown in Table 3, there was no significant difference between previously hyperactive and controls (*F* = 0.63; *df* = 1, 62; *p* = .43), and those identified as hyperactive in childhood were no less likely to show a “good” level of satisfaction, χ^2^(1) = 0.41, *p* = .84.

### Comparison Between Hyperactive and Nonhyperactive Adults

According to the *DSM-IV* diagnostic ratings at age 27 years, 22.5% of all formerly hyperactive participants met criteria for ADHD in adulthood. In accordance with the second aim of the study, the participants with adult ADHD and those without hyperactivity were compared, and results are in Table 4. Those with adult ADHD reported poorer negotiation skills (*F* = 4.89; *df* = 1, 63; *p* < .05) and less satisfaction in partner and other intimate relationships (*F* = 7.03; *df* = 1, 63; *p* < .05), compared with the participants without hyperactivity. In contrast, both groups did not differ with regard to the level of satisfaction with friendships or support received from the family, and both groups were similar to the controls.

**Table 4.** Comparison of Relationship Satisfaction Levels Between Participants Who Had ADHD at Age 27 and Participants Who Had Subthreshold Levels of Hyperactivity at Age 27.

	Adult ADHD	Not adult ADHD	*p*
Satisfaction with friends	2.79 [0.802]	2.64 [0.954]	.589
Family support	2.15 [0.689]	1.92 [0.896]	.382
Ability to negotiate	3.36 [0.929]	2.80 [0.850]	.03
Satisfaction in partner and intimate relationships	3.93 [0.917]	3.03 [1.197]	.01

Comparisons between those hyperactive only in childhood and those with hyperactivity in childhood and ADHD in adulthood were also carried out. Those identified as hyperactive in childhood with ADHD in adulthood did not differ significantly from those hyperactive in childhood only and from controls in their level of ability to negotiate (*F* = 3.50; *df* = 1, 38; *p* = .07), satisfaction with relationships (*F* = 3.47; *df* = 1, 38; *p* = .07) or family support (*F* = 3.09; *df* = 1, 37; *p* = .09). These results might suggest that persistence of ADHD could be linked to adverse social outcomes, but the number of participants is too small to base conclusions.

Finally, to examine whether antisocial behavior had an influence on the associations, a global measure of antisocial behavior was created. The number of offenses found in criminal records, self-reported aggressions, and fights and use of weapons were added to create this measure. Using an ANCOVA, with hyperactivity levels in adulthood as predictor, antisocial behavior as covariate, and relationship satisfaction levels as dependent variables, antisocial behavior was not found to be significantly related to intimate partner relationships, *F*(1, 61) = 0.43; negotiation skills, *F*(1, 61) = 0.60; family support, *F*(1, 61) = 1.4; or friendships, *F*(1, 61) = 0.72, and the predictiveness of hyperactivity was unaltered.

## Discussion

This study shows that adult ADHD is associated with difficulties in social relationships, especially intimate partner relationships, and with poor negotiation skills. These findings generally corroborate and extend those of previous studies suggesting that children with ADHD are at an increased risk for social dysfunction in later life (Barkley et al., 2006). Adults with ADHD have difficulties in romantic relationships (Halversted, 2002), more psychological maladjustment (Murphy & Barkley, 1996), and impairment in a number of areas of functioning (Wilens & Dodson, 2004) has been documented. Previous follow-ups of our sample also support the view that persistence of hyperactivity is a key influence on adult psychopathology and poor outcome (Taylor et al., 1996).

Another interesting finding from this study was that the participants who had been hyperactive in childhood, but did not have ADHD in adulthood, were not obviously less satisfied with their social relationships than those who had never been hyperactive. The rather negative picture of the social outcome of children referred to specialist clinics, and diagnosed with ADHD, should not necessarily be generalized to the broader range of hyperactive children in the community. Most hyperactive children may have a different adult outcome from that reported in clinic-referred ADHD children (Barkley, Fischer, Smallish, & Fletcher, 2004; Mannuzza, Klein, Bessler, Malloy, & Lapadula, 1998).

It is possible that the method of self-report may have underestimated the degree of impairment in relationships if people with hyperactivity lack insight into any problems that may be present. This explanation would not, of course, diminish the significance of the differences we found in adults who showed diagnosable ADHD.

The question arises why milder degrees of childhood hyperactivity are not associated with relationship dissatisfaction in adulthood in most cases. This may be due to the overall decrease of hyperactivity from childhood to adulthood. In a previous follow-up of this sample, hyperactivity was found to diminish between the ages of 7 and 17 years (Taylor et al., 1996). Consistent with this, Biederman, Mick, and Faraone (2000) reported that hyperactivity symptoms declined at a higher rate than inattention symptoms, and Asherson’s (2009) meta-analysis on the prevalence of adult ADHD shows that a high proportion of children grow out of the disorder—either due to maturational changes in brain function and self-regulation or because of learned coping skills. Moreover, adult life offers more opportunities to choose an environment that is more suitable for someone who has ADHD. Hyperactive adults may find supportive friends and partners who can help them improve poor self-discipline, overreacting to frustration, difficulty in self-organization, and establishing and keeping routines.

The association of hyperactivity and antisocial behavior has been documented in clinic-referred samples (Barkley et al., 2004; Mannuzza, Klein, Bessler, Malloy, & Lapadula, 1993). However, antisocial behavior is known to be frequently associated with childhood hyperactivity and referral to services (Woodward, Dowdney, & Taylor, 1997).

Therefore, it may only be possible to examine the impact of hyperactivity accurately in epidemiological studies. To our knowledge, there exist only two larger epidemiological studies. Bussing, Mason, Bell, Porter, and Garvan (2010) screened 1,615 children in a school district and followed them for 8 years. In their study, childhood ADHD was found to be associated with persistence in nearly half of the sample, together with an increased risk for comorbidity, functional impairment, and reduced quality of life. Subthreshold ADHD increased the risk for grade retention, but social relationships were not analyzed.

The Christchurch Health and Development Study (Fergusson, Horwood, & Ridder, 2007) examined the mediating role of conduct problems in a general population sample and found that any association between early attentional problems and the adverse outcome of substance misuse was mediated via the association between conduct and attentional problems. By contrast, our epidemiological data do not suggest that antisocial behavior determines the relationship between ADHD and social dysfunction.

It has to be noted that none of the children in our sample had received medication for symptoms of ADHD. At that time, and in this part of London, the diagnosis was not used and medication was not prescribed to any of the children. If a referral to a mental health service was made, then the offer was of family therapy, which was usually unacceptable to the parents. Hence, the course of hyperactivity was not confounded by the effects of specific therapeutic intervention. It is uncertain whether medication is a protective factor in the longer term, and more research is needed to clarify its effect on the long-term course of ADHD symptoms. Our results do not support the treatment of young people with subdiagnostic levels of hyperactivity. Neither do they provide evidence for screening for hyperactivity in the general population. The majority of untreated children in our sample appeared to have a positive outcome regarding social relationships. However, reverse causality cannot be ruled out. Relationship problems may influence emotional well-being and increase the likelihood of affected individuals continuing to display high levels of hyperactivity. Whether this can happen in adulthood is not known. To date, little is known about the long-term developmental course of hyperactivity and accurate knowledge about factors involved in negative and positive outcome is needed. Future well-designed, long-term studies should contribute to gaining a better understanding of these factors, which in turn would help to define high-risk groups and eventually lead the way to new targets for intervention.

### Limitations of the Study

This study has controlled for antisocial and emotional problems, but as in other long-term epidemiological studies there remains the possibility for other unmeasured, uncontrolled confounding factors. The rather small sample size entails limited power, and the attrition rate of around 30% might have had an effect on the validity of the results. Social relationships were documented through self-reports, which could have exaggerated, or understated, the associations with hyperactivity.
